# Extra Limites

**Published:** 1836-07-01

**Authors:** 


### EXTRA-LIMITES.

### NOTES OF AN EPIDEMIC YELLOW FEVER, WHICH OCCURRED AT ANTIGUA IN THE LATTER PART OF 1835.

By JOHN FURLONG, M. D., M. R. C. S.
ASSISTANT SURGEON-GENERAL OF ANTIGUA.

Antigua has had an exemption from yellow fever 19 years; at least as far as I can ascertain, no case of fever, with decided black vomit, has been observed by civil practitioners since 1816 till within the last three months, when an alarm of its appearance was sounded, and its inhabitants thrown into dismay, their long immunity having almost lulled them into believing, that this scourge of the Tropics had for ever left their shores.

The epidemic with which Antigua has been visited was severe, though brief. It is my intention to give a succinct sketch of the fever—its probable cause, symptoms, treatment, &c. The island, prior to August, was unusually dry and hot; on the 12th of that month, it witnessed one of the severest hurricanes in the memory of the oldest inhabitants, accompanied with much rain, succeeded by extremely hot and dry weather—so much so, that it was generally remarked that part of September, and all October, were the driest months observed for many years. The violence of the storm occasioned an almost complete stripping of the leaves of the trees, giving them the seared and naked appearance of Autumn; thus causing a great deposition of vegetable matter, and no doubt mixed with myriads of animalculæ destroyed in the gale, both well saturated with the rains which fell with and succeeded the atmospheric conflict. This state was followed up for some weeks by great heat and drought. Such being the fact, no one can be surprised at the generation of malaria, and that in a highly concentrated state, and from such, unquestionably, the epidemic took its origin. The disease was almost exclusively confined to St. John's and English Harbour, and the cases which occurred in other parts of the island could always be traced to the sick having visited one or the other. Perhaps there is no tropical town more favourably situated for the production of malaria than

St. John's ; it is surrounded on three parts by gently rising hills, and, consequently, is a receptacle for the debris, and all kinds of malaria-engendering matter, from the surrounding acclivities. The fever broke out in the very part of the town one, à priori, would have imagined—at the bottom called the Point, where the washing from the upper parts of St. John's are accumulated ; and that locality is, at most favourable times, justly considered a hot-bed of malaria. The fever lasted from September till the latter part of November, a few sporadic cases occurring subsequently, and mostly in the country. The disappearance of the fever was as sudden as its invasion was unexpected, and its absence is no doubt to be ascribed to some genial showers which fell about that time, and have since continued. The fever was ushered in with shiverings, or a sense of cold creeping down the back, —shuddering or horripilation—pain of back, neck, and hips—irritability of stomach—headach, in some cases, very severe, in others, amounting only to a sense of tension of eyeballs and forehead, with a difficulty of elevating the eyes—tongue dry and furred—pulse, for first 24 hours, full and quick (I confess I never felt it strong or hard), and in bad cases, after 24 or 30 hours, symptoms became decidedly *typhoid*, with prostration of strength, collapsed features, listless eye, low delirium, floccitatio, pulse soft, and seldom above 80, passive hæmorrhages from gums, yellow skin, ecchymosis, black vomit, &c. and in one case, blood exuded from the pores of the skin at the side of the nose. I had only the opportunity of witnessing three post-mortem examinations: in these, the gastro-intestinal mucous membrane was in a state of great congestion—not arising from inflammation, but from passive congestion, occasioned, in my opinion, by an increased *tenuity* of the blood, whereby it gained admittance into vessels which, in its normal state, do not carry it. That this condition was not inflammatory, I judged from the symptoms in the living, and the appearance in the dead. In a plethoric young Scotch gentleman, who had passive hæmorrhage from the gums, and who recovered, I pressed the abdomen in every possible way, without giving any uneasiness, and he felt none. I have no doubt that his gastro-intestinal mucous coat was in the state above-mentioned, or approaching thereto. The rest of the viscera did not betray much deviation from health; in one of the cases, the liver seemed paler than usual, and the stomach contained a pint of thin blood, and some was extravasated among the fibres of the left pectoral muscle. During the epidemic, there was a great hæmorrhagic tendency; most females, when attacked, had a flow of blood from the uterus, and in the case of a young lady whom I attended, to such an extent, that I was obliged to prescribe acetate of lead, and order cold lotions to the pudenda, &c. and I have no doubt her death was expedited by the loss of blood. Another young lady who had been obstructed for nearly four months, occasioned by a wetting, was attacked with fever, and on the third day of her indisposition, was seized with menorrhagia. She had been under a course of aloetic pills and the semicupium without benefit. The blood from the gums had the appearance of treacle and water; the crasis of the blood seemed diminished, and its fluidity evidently increased ; could it have been possible to transport one of the cases, after 36 hours, to a hospital in England (as some of my medical friends agreed with me), an English physician would have called it the malignant fever of Huxham. The fever seized *all colours*—long residence in the country was no exemption ; black, white, and coloured were carried off by the epidemic, and some by a second invasion. Blacks became yellow, as far as it could be observed on the skin, but was well marked in the eye. One gentleman, who had been nearly 20 years on the island, died with black vomit. The fever showed all grades, from intermittent—remittent—to concentrated yellow fever, proving a common origin, the type varying according to susceptibility, or acclimated state of the individual, though not without exceptions. With regard to treatment, it was soon found that the fever did not bear bleeding; it was fairly tried by some, and given up. I never bled one of my patients, and lost two out of 50 or 60 of all colours. I witnessed no symptom warranting the use of the lancet, though I would be far from stating that such might not have occurred in the patients of others. Calomel was the sheet-anchor, ten or fifteen grains, followed up in two hours by a dose of sulph. mag. et mag., and then every second hour after, while feverish, 3, 5, or 10 grains were given in a pill, combined with $\frac{1}{4}$ of a grain of opium and effervescing draughts, with nitre and sweet spirit of nitre between; sinapisms to scrob. cordis, to check vomiting, were directed with good effect; if headach was severe—the hair was thinned or shaved and cold applied, or cold douche, and sometimes it was necessary to blister the neck before relief was obtained. A tepid bath and warm pediluvia were occasionally used; the principle on which they were ordered is obvious—they tended much to relieve internal congestion. As soon as the gums were affected, the patient was generally safe, and quinine was ordered in two-grain doses every two hours. Such is a brief but faithful outline of the epidemic.

The different opinions of writers on tropical yellow fever are surprising, and well calculated to confound the inexperienced. One tells you your sole dependance is on the lancet—it is a sine quâ non; another states, after crossing the Tropics, throw your lancets overboard! Can such conflicting statements be reconciled? I think they may. It is well known that epidemics differ, arising from season and atmospheric constitution. The epidemic I have described occurred in the fall of the year, when all organic life appears depressed, as Nature herself points out; fevers at this time of the year assume an asthenic character. It was an observation of Sydenham, Gregory, and others, that Autumnal epidemics do not bear bleeding or debilitating remedies. Fever, occurring in the Spring or Summer months, puts on a phlogistic type, at which time, bleeding and other powerful antiphlogistics are indicated, and necessary to the salvation of the patient, facts which I have many a time witnessed. It is from not marking the different seasons, that we find such conflicting views of yellow fever by authors: one sees and describes a vernal epidemic—bleeding is the sheet-anchor; true:—Another Autumnal—the lancet not indicated; true also. In this way I would endeavour to reconcile such antagonising views, though something, also, is due to the malignancy of the occasional cause, longer or shorter exposure thereto, and other circumstances, which will suggest themselves to the medical reader. The black vomit is evidently altered blood; there can be no doubt that the patient who has hæmorrhage from his gums, has the same condition of the gastro-intestinal mucous coat, for many passed malæna-looking stools, the blood in the stomach, &c. passing downwards, and not appearing as black vomit. I mentioned a patient in whose stomach a pint of blood was found, which, had it come up, would have been black vomit. Most individuals who had no formal attack of the fever, were nevertheless far from being well; they complained of general malaise, the gastro-intestinal and other secretions were vitiated, evinced by foul-coated tongue. I had a slight attack of the epidemic, and it was three weeks after the fever left me before my tongue became clean. Every *medical man* in St. John's, with one exception, was attacked, and all remarked the same obstinately-furred condition of the tongue. We had inhaled and were inhaling a poison; the susceptible had fever, while those less susceptible suffered also, though without formal fever, just as an individual, accustomed to take opium daily, feels a little stupid at an increased dose, which, indulged in by one unused thereto, would occasion alarming symptoms, if not death. Calomel acted, I conceive, in this fever, not so much as an anti-inflammatory, as by promoting the secretions, and restraining internal congestions within safe limits—equalising the circulation, and thus removing the overwhelming load which threatened destruction to the patient. I offer these remarks as a sketch only of a severe fever; but sufficient, I hope, has been advanced to give an idea of the features of the enemy with which we had to contend.

With regard to contagion, I may venture to say that it meets with no advocates in this Island, all being true anti-contagionists. During the greater part of the epidemic, I was in medical charge of the troops stationed in St. John's—detachments of the 36th, 74th, and 1st West India regiments. I immediately had the zealous co-operation of Captain Murray of the 36th, and Lieutenant O'Brien of the 74th, in issuing an order prohibiting the men going into town, and the result was, that, with exceptions I shall mention, three or four slight cases only occurred. The barracks at St. John's are directly to the east of the town. A soldier of the 36th regiment came into the barracks on leave from the Ridge (English Harbour), where the epidemic was raging among the garrison; he took fever, or rather brought it with him, and died without spreading it. Staff assistant surgeon Dr. Hopkins relieved me at the close of the epidemic: two men of the 74th regt. were brought over to St. John's barracks, from the neighbouring Island of Barbadoes (where there is a detachment), both with malignant yellow fever—one dying in seven hours after his landing, and the other in five days, with passive hæmorrhages, both most assiduously and affectionately attended by their comrades (sleeping in the same barrack-room, there being no hospital in St. John's); and yet no other case occurred!! Does this look like contagion? I attribute the immunity of the men to the order, restricting them to barracks.

ST. JOHN'S, ANTIGUA,
30*th Dec.* 1835.

